# Outpatient step-up dosing of bispecific antibodies in relapsed or refractory multiple myeloma: an oncology nursing framework for monitoring and supportive care

**DOI:** 10.3389/fonc.2026.1859755

**Published:** 2026-06-09

**Authors:** Yanfeng Song, Xiaofei Zhou, Lina Wang

**Affiliations:** Department of Oncology, The First Hospital of Jilin University, Changchun, China

**Keywords:** bispecific antibodies, bone marrow microenvironment, cytokine release syndrome, immune effector cell-associated neurotoxicity syndrome, multiple myeloma, oncology nursing, outpatient step-up dosing, supportive care

## Abstract

Bispecific antibodies have expanded treatment options for relapsed or refractory multiple myeloma, but treatment initiation remains operationally challenging because product-specific step-up dosing concentrates the highest risk of early immune-mediated toxicity. This structured narrative review synthesizes current prescribing information, clinical safety reports, expert guidance, implementation literature, and nursing-relevant supportive-care evidence for ambulatory step-up dosing. We revised the framework to move beyond a label-based summary and conceptualize outpatient initiation as a biologically informed, resource-stratified, and failure-aware model. Approved agents differ in target antigen, route of administration, step-up schedule, premedication, cytokine release syndrome (CRS) timing, monitoring requirements, and long-term toxicity patterns; these differences should translate into different observation windows, home-surveillance instructions, and escalation thresholds. Patient selection should integrate clinical stability, disease burden, immune reserve, baseline neurologic status, caregiver reliability, geography, communication capacity, and institutional rescue capability. The bone marrow microenvironment, T-cell fitness, tumor adaptation, antigen heterogeneity, and cumulative immune injury from prior therapies may influence both response and toxicity, supporting a translational approach to outpatient risk assessment. We propose practical decision matrices for product-specific monitoring, candidate assessment, CRS-infection dual-track fever evaluation, neurotoxicity detection, failure-mode mitigation, and continuous program evaluation. Outpatient step-up dosing should therefore be considered a selective high-acuity care model rather than a routine efficiency strategy. Prospective validation of eligibility criteria, remote monitoring, nursing-sensitive outcomes, and resource-stratified pathways is urgently needed.

## Introduction

Multiple myeloma (MM) is a plasma cell malignancy characterized by recurrent cycles of response, clonal evolution, immune dysfunction, and relapse. Despite substantial advances with proteasome inhibitors, immunomodulatory agents, anti-CD38 monoclonal antibodies, autologous stem-cell transplantation, and cellular therapies, many patients eventually develop relapsed or refractory multiple myeloma (RRMM) and require additional treatment options with distinct mechanisms of action (1, 2).

T-cell-redirecting bispecific antibodies (BsAbs) have become an important therapeutic class for RRMM. By engaging CD3 on T cells and a tumor-associated antigen on myeloma cells, most commonly B-cell maturation antigen (BCMA) or G protein-coupled receptor class C group 5 member D (GPRC5D), these agents redirect endogenous cytotoxicity toward malignant plasma cells. The therapeutic mechanism is inseparable from immune activation, and the toxicity profile includes CRS, immune effector cell-associated neurotoxicity syndrome (ICANS), cytopenias, infection, hypogammaglobulinemia, and, for GPRC5D-directed therapy, characteristic oral, taste, skin, nail, and weight-related adverse events (3–6).

To reduce the risk of severe early toxicity, currently approved BsAbs use product-specific step-up dosing during treatment initiation. At the same time, health systems face increasing pressure to reduce inpatient utilization and to deliver complex cancer therapies in ambulatory environments. These forces have created interest in outpatient step-up dosing for selected patients. However, outpatient initiation should not be understood as a simple relocation of inpatient drug administration. It is a high-acuity care model in which drug-specific toxicity kinetics, patient-level biologic vulnerability, caregiver performance, and institutional rescue capacity must align (7–11).

The original version of this review focused primarily on practical nursing workflows. In this revised manuscript, we strengthen the analytical framework by integrating clinical operations with underlying disease biology and by explicitly addressing implementation variability, failure points, uncertainty, and continuous quality improvement. Our central premise is that safe ambulatory initiation requires a biologically informed, resource-stratified, and failure-aware nursing model rather than a generic outpatient checklist.

## Scope and structured narrative review approach

This review is narrative in design because the available evidence is heterogeneous and includes prescribing information, single-arm clinical trials, expert consensus, infection-management recommendations, institutional implementation reports, and nursing-practice literature. Nevertheless, to improve transparency and reproducibility, we used a structured narrative approach rather than an unbounded descriptive summary (12, 13).

Targeted searches of PubMed/MEDLINE and CINAHL were performed for literature available through March 2026. Search domains included: (1) multiple myeloma or relapsed/refractory multiple myeloma; (2) bispecific antibody, T-cell redirection, teclistamab, elranatamab, talquetamab, linvoseltamab, BCMA, GPRC5D, and CD3; (3) step-up dosing, outpatient, ambulatory, hospitalization, monitoring, triage, and remote monitoring; (4) CRS, ICANS, neurotoxicity, infection, hypogammaglobulinemia, cytopenia, and supportive care; and (5) nursing, patient education, caregiver, handoff, teach-back, and implementation outcomes. Reference-list screening and current regulatory documents were also used to identify clinically relevant sources.

Sources were included when they addressed approved or late-stage BsAbs in RRMM, early toxicity monitoring, outpatient or hybrid step-up implementation, infection prevention, neurotoxicity or CRS management, agent-specific supportive care, patient education, nursing workflow, or implementation outcomes. Sources were deprioritized or excluded if they were preclinical-only, unrelated to MM, focused exclusively on chimeric antigen receptor T-cell therapy without transferable toxicity-management principles, or lacked sufficient clinical or operational detail. Conference abstracts were not used as primary evidence when peer-reviewed full-text reports were available.

Evidence was interpreted according to a predefined hierarchy. Current prescribing information was treated as the primary source for label-based dosing, premedication, and monitoring requirements. Prospective trial reports were prioritized for product-specific safety patterns. Consensus recommendations and expert guidance were used to interpret toxicity grading, infection prevention, and supportive care. Real-world implementation reports and nursing literature were used to translate evidence into ambulatory pathways. When data were discrepant, we prioritized label requirements for operational safety, then described variability across studies instead of presenting a single pooled estimate. Because no formal dual-reviewer screening, meta-analysis, or structured risk-of-bias assessment was performed, the manuscript should be interpreted as a structured practice-oriented synthesis and hypothesis-generating framework rather than a systematic review or universal standard.

## Conceptual model: outpatient step-up dosing as a high-acuity, biologically informed care system

A useful outpatient framework must answer more than whether a BsAb can be administered outside the hospital. It must determine whether the patient, caregiver, drug, clinic, and emergency system together can identify and reverse early toxicity before physiologic deterioration becomes irreversible. We therefore conceptualize outpatient step-up dosing as a dynamic care system with three interdependent pillars.

The first pillar is product-specific immune-toxicity kinetics. Route of administration, step-up schedule, premedication, target antigen, and typical timing of CRS influence how long patients require direct observation, when scheduled calls should occur, and how conservative the threshold for admission should be. The second pillar is patient-level biological vulnerability, including disease burden, tempo of progression, bone marrow reserve, immune status, prior treatment exposure, baseline neurologic function, frailty, and infection risk. The third pillar is rescue capacity, defined by nursing expertise, after-hours access, emergency department preparedness, tocilizumab and corticosteroid availability, inpatient-bed access, intensive-care support, transport reliability, and caregiver readiness (14, 15, 19, 20).

This model deliberately rejects two oversimplifications. The first is that outpatient initiation is inherently safer once grade 3 or higher CRS is uncommon. Even grade 1 fever may be dangerous when it is misclassified, reported late, or occurs in a patient with neutropenia and limited transport. The second is that outpatient feasibility is determined mainly by institutional preference. In practice, the same product and patient can be suitable in a tertiary center with 24-hour hematology coverage and unsafe in a setting without rapid escalation.

## Bone marrow microenvironment, immune architecture, and translational relevance to outpatient risk

The clinical operations of outpatient step-up dosing are inseparable from MM biology. The bone marrow microenvironment is not a passive site of disease but a dynamic interface where tumor adaptation, stromal support, immune suppression, angiogenesis, cytokine release, and therapeutic pressure coexist. Solimando and colleagues emphasized that drug resistance in MM reflects both intrinsic tumor mechanisms, including clonal heterogeneity, drug efflux, altered drug targets, inhibition of apoptosis, and increased DNA repair, and extrinsic mechanisms mediated by bone marrow stromal cells, adipocytes, osteoclasts, osteoblasts, endothelial cells, immune cells, cell adhesion, and soluble factors (42).

This concept is directly relevant to BsAbs. T-cell redirection can overcome some forms of tumor-intrinsic resistance by recruiting cytotoxic effector function, but it remains dependent on antigen expression, T-cell fitness, inflammatory reserve, and the suppressive structure of the marrow niche. Heavily pretreated patients may have reduced marrow reserve, lymphopenia, T-cell exhaustion, hypogammaglobulinemia, and impaired vaccine responsiveness. Myeloma cells and their microenvironment can also promote T regulatory cells, myeloid-derived suppressor cells, inhibitory checkpoint expression, cytokine networks, and adhesion-mediated survival signals that may shape both efficacy and toxicity (21, 24, 42).

The reviewer correctly noted that resistance can begin during precursor states such as monoclonal gammopathy of undetermined significance and evolve through progressive niche remodeling. For outpatient care, this biology does not yet translate into a validated toxicity score. It does, however, justify more explicit documentation of disease burden and immune reserve before ambulatory initiation. High marrow involvement, rapidly rising light chains, extramedullary disease, elevated lactate dehydrogenase, renal dysfunction, baseline cytopenias, recent infections, profound hypogammaglobulinemia, and prior exposure to anti-CD38 or BCMA-directed therapy should not be viewed as background descriptors only. They are plausible markers of reduced physiologic and immunologic reserve that may lower tolerance for even low-grade early toxicity.

Accordingly, the revised framework treats outpatient eligibility as a translational decision. Clinical logistics remain essential, but they should be interpreted together with disease biology, immune architecture, and cumulative treatment history. This approach does not replace future predictive modeling; rather, it creates standardized domains that can be prospectively captured and tested.

## Product-specific operational differences and decision implications

Approved BsAbs share a T-cell-redirecting mechanism but cannot be managed through a single generic outpatient pathway. Their differences in target antigen, route, step-up schedule, premedication, timing of CRS, and non-CRS toxicity patterns have direct nursing implications (4–10, 16–18). The purpose of **Table 1** is therefore not only to summarize label information, but to translate product-specific characteristics into operational decisions.

**Table 1:** Product-specific operational decision matrix for bispecific antibody step-up dosing in RRMM .

Agent and route	Step-up and premedication features	CRS timing and label-based monitoring	Decision implications for outpatient nursing
Teclistamab (BCMA × CD3; subcutaneous)	Step-up dose 1: 0.06 mg/kg; step-up dose 2: 0.3 mg/kg; first treatment dose: 1.5 mg/kg, then weekly with possible interval adjustment in sustained responders. Premedication per label includes corticosteroid, antihistamine, and acetaminophen before step-up doses.	Median CRS onset in pivotal reporting was approximately 2 days after the most recent dose. Current label-based monitoring requirements include prolonged observation/hospitalization during early step-up dosing; local requirements must be verified.	Delayed CRS timing means that a clinic-only observation period is insufficient unless paired with reliable caregiver observation, daily follow-up during the risk window, written fever action plans, and rapid return-to-care pathways. Patients with poor communication, remote residence, high disease burden, or limited caregiver support are better suited to inpatient or hybrid initiation.
Elranatamab (BCMA × CD3; subcutaneous)	Step-up dose 1: 12 mg; step-up dose 2: 32 mg; treatment dose: 76 mg, then weekly with label-based interval extension in responders. Premedication is required before the first three doses according to prescribing information.	Median CRS onset was approximately 2 days after the most recent dose. Label-based hospitalization or close monitoring applies during early doses, with product-specific durations.	The first treatment week contains repeated risk transitions. Nursing workflows should schedule reassessment after each dose, verify response-based interval changes only after milestones are met, and avoid assuming that tolerance of the first step-up dose ensures safety after later doses.
Talquetamab (GPRC5D × CD3; subcutaneous)	Weekly and every-2-week step-up schedules are available. Premedication before step-up doses includes corticosteroid, antihistamine, and acetaminophen per label.	Median CRS onset was approximately 27 hours after the last dose in pivotal reporting. Label-based monitoring includes prolonged observation/hospitalization for step-up doses. Oral, taste, skin, nail, and weight-related events may persist after the highest CRS-risk period.	Outpatient feasibility must consider both acute immune toxicity and medium-term functional toxicity. Nurses should initiate early nutrition, oral care, dermatologic, nail, and weight surveillance rather than waiting for severe intake decline or quality-of-life impairment.
Linvoseltamab (BCMA × CD3; intravenous)	Step-up doses include 5 mg and 25 mg before full-dose treatment at 200 mg according to the label reviewed. Pretreatment medications include acetaminophen, antihistamine, and corticosteroid-equivalent prophylaxis per label.	Median CRS onset was reported at approximately 11 hours from the end of infusion after the most recent dose. Current label-based monitoring includes observation/hospitalization after early step-up doses.	Intravenous administration and earlier CRS timing shift emphasis toward infusion-day assessment, overnight surveillance, and rapid differentiation of CRS from infusion-related reactions. Discharge after infusion requires explicit overnight monitoring and return instructions.

This table summarizes selected operational features from prescribing information and pivotal safety reports available to the authors at the time of revision. Institutions must verify the most current local regulatory label before implementation because dosing, monitoring requirements, and indications may change.

Subcutaneous agents such as teclistamab, elranatamab, and talquetamab may produce delayed CRS after patients have left direct observation, making home surveillance, after-hours triage, and caregiver education critical. In contrast, an intravenous agent such as linvoseltamab may concentrate risk closer to infusion-day or overnight observation and requires nurses to distinguish CRS from infusion-related reactions. Target antigen also matters. BCMA-directed therapy is associated with deep plasma-cell and humoral immune effects, whereas GPRC5D-directed therapy adds persistent oral, taste, skin, nail, and nutritional toxicities that require longitudinal supportive care (9, 18, 24, 31, 32).

## Patient selection: from qualitative checklist to risk-domain assessment

Patient selection is the central safety decision in outpatient step-up dosing. The revised framework distinguishes between eligibility, modifiable risk, and feasibility. Eligibility addresses whether the patient is physiologically stable enough to start therapy outside a conventional inpatient pathway. Modifiable risk addresses problems that can be corrected before dosing, such as uncontrolled infection, inadequate education, missing caregiver coverage, or lack of transportation. Feasibility addresses whether the local care system can respond when toxicity develops (14, 15, 20, 21).

The variables in **Table 2** are not presented as a validated prediction score. No prospective model currently defines which RRMM patients can safely receive all step-up doses in an outpatient setting. However, standardized documentation of measurable risk domains can reduce selective decision-making, make multidisciplinary discussions more explicit, and create a dataset for future validation. In practice, the presence of multiple red flags should lower the threshold for inpatient or hybrid initiation even when each factor alone appears manageable.

**Table 2:** Measurable risk-domain framework for outpatient step-up dosing suitability .

Domain	Suggested measurable assessment	Red flags	Operational implication
Clinical stability	Temperature trend; blood pressure; heart rate; oxygen saturation; active or resolving infection; current antimicrobials; new oxygen requirement; hydration; renal function.	Unexplained fever, hemodynamic instability, new hypoxia, sepsis concern, uncontrolled infection, or inability to maintain oral intake.	Delay dosing, treat reversible problem, or initiate in an inpatient setting.
Disease biology and tumor burden	Bone marrow plasma-cell burden; rapidly rising M-protein or free light chains; LDH; extramedullary disease; circulating plasma cells when available; renal dysfunction; pace of progression.	Rapidly progressive high-burden disease, aggressive extramedullary disease, renal compromise from high light-chain burden, or high LDH suggesting aggressive biology.	Consider inpatient or hybrid initiation, intensified monitoring, and lower threshold for early admission. Capture variables prospectively for future risk modeling.
Immune reserve and infection susceptibility	Absolute neutrophil count; lymphocyte count; IgG level; recurrent infection history; prior anti-CD38 therapy; prior BCMA-directed therapy or CAR T-cell therapy; steroid exposure; vaccination status.	Severe neutropenia, profound hypogammaglobulinemia, recent serious infection, recurrent opportunistic infection, or cumulative immune suppression.	Plan prophylaxis, consider IVIG according to local criteria, intensify fever work-up, and avoid outpatient initiation if infection rescue capacity is limited.
Baseline neurologic status	Orientation; handwriting sample; ICE or structured cognitive screen; history of seizure, stroke, delirium, neuropathy, or cognitive impairment; sedating medications.	Delirium, severe cognitive impairment, inability to establish baseline, unreliable symptom reporting, or caregiver unable to recognize change.	Require inpatient initiation or enhanced neurologic monitoring; document baseline handwriting and cognition before each step-up dose.
Frailty and physiologic reserve	ECOG performance status or frailty index; falls; baseline oxygen need; nutritional status; weight loss; sarcopenia; comorbid cardiac, pulmonary, renal, or hepatic disease.	Marked frailty, dependence for basic activities, recent falls, baseline oxygen requirement, severe malnutrition, or limited ability to tolerate hypotension or fever.	Favor inpatient or hybrid initiation; involve nutrition, rehabilitation, social work, and palliative/supportive care as needed.
Caregiver reliability	Dedicated caregiver availability during highest-risk windows; ability to remain with patient overnight; capacity to measure temperature; comprehension of CRS/ICANS red flags; backup caregiver.	No reliable caregiver, caregiver fatigue, inability to provide continuous observation, or inability to perform teach-back.	Do not use a fully outpatient model unless reliable coverage can be arranged.
Geography and transport	Travel time to treating center or capable emergency department; night-time transport; weather or rural barriers; temporary lodging options.	Remote residence, unsafe night travel, lack of vehicle, or inability to return promptly.	Use inpatient, hybrid, or near-center lodging strategy; pre-identify emergency department and transfer route.
Communication and health literacy	Phone access; language needs; literacy; ability to use symptom diary or remote monitoring; understanding of whom to call after hours.	No phone access, language discordance without interpreter plan, inability to understand fever action plan, or previous delayed reporting.	Provide translated materials, teach-back, wallet card, scheduled calls, and direct escalation script; inpatient initiation if communication remains unreliable.
Institutional rescue capacity	24/7 hematology coverage; trained triage nurses; tocilizumab and corticosteroid availability; emergency department protocol; inpatient/ICU backup; handoff process.	No after-hours pathway, emergency department unfamiliarity, lack of rapid transfer, limited access to CRS-directed therapy, or recurrent bed shortages.	Outpatient step-up dosing should not proceed as a default; build infrastructure or use inpatient initiation.

The framework standardizes assessment but has not been prospectively validated. Local programs should record these domains and evaluate their association with CRS, ICANS, infection, emergency visits, unplanned admission, and treatment delays.

## Monitoring during and after step-up dosing

Monitoring spans pre-dose verification, immediate post-dose observation, structured transition to home or post-discharge surveillance, and after-hours escalation. The revised framework emphasizes temporal alignment: the intensity and timing of monitoring should correspond to the product, the dose, the patient’s baseline risk, and the local system’s ability to rescue toxicity (7–10, 19, 20).

Before each step-up dose, nurses should confirm the absence of new fever, infection symptoms, progressive dyspnea, hypotension, altered mentation, or other clinical instability. Laboratory review should include the complete blood count trend, renal and hepatic function as locally required, and markers relevant to infection or tumor burden when clinically indicated. Nurses should verify required premedications, confirm that emergency medications and escalation contacts are available, document baseline neurologic status, and re-confirm caregiver and transportation arrangements for the next risk window.

During direct observation, serial measurement of temperature, heart rate, blood pressure, respiratory rate, oxygen saturation, and neurologic function is essential. Observation intensity should increase when symptoms such as rigors, myalgia, headache, tachycardia, dizziness, new oxygen need, confusion, tremor, or unusual somnolence emerge. A patient who appears stable at the end of a clinic visit may still be within the expected CRS window; therefore discharge should occur only after the next monitoring steps are explicit.

Home surveillance should be time-limited, written, and rehearsed. Patients and caregivers should know the required temperature-check schedule, how to document symptoms, what threshold constitutes fever, which neurologic or respiratory symptoms require urgent contact, and whether antipyretics are prohibited unless directed. Scheduled telephone calls or remote monitoring can improve oversight but should not replace the ability to perform urgent in-person assessment.

## Failure-aware outpatient monitoring

A major revision in this manuscript is the explicit recognition that outpatient pathways fail not only because toxicity occurs, but because recognition, communication, or escalation fails. **Table 3** summarizes common failure modes and mitigation strategies.

**Table 3:** Failure modes in outpatient step-up dosing and mitigation strategies .

Failure point	Potential consequence	Mitigation strategy
Delayed recognition of fever or rigors	Progression from early CRS or infection to hypotension, hypoxia, sepsis, or late admission.	Teach-back of fever threshold; written fever action card; scheduled calls after step-up doses; caregiver instructed to wake patient for checks during the risk window if locally required.
Self-administration of antipyretics before contacting team	Masked fever, delayed grading, and delayed infection or CRS work-up.	Explicit discharge instruction: do not treat suspected post-dose fever with over-the-counter antipyretics unless instructed by oncology team.
Caregiver variability or fatigue	Symptoms occur overnight without reliable observation; patient under-reports confusion or dyspnea.	Confirm caregiver availability before each dose; identify backup caregiver; avoid fully outpatient model if continuous observation cannot be guaranteed.
Off-hours communication breakdown	Patient calls wrong number, reaches non-specialist advice line, or receives delayed call-back.	Provide single 24/7 contact pathway, escalation script, direct hematology backup, and documentation of last dose and toxicity risk in the electronic record.
Emergency department unfamiliarity with BsAbs	Fever misclassified as routine infection or, conversely, infection dismissed as CRS; delayed tocilizumab, antibiotics, or admission.	Use wallet card, ED protocol, standardized handoff template, and direct clinician-to-clinician communication.
Long travel distance or unsafe night transport	Triage recommends return but patient cannot reach care promptly.	Arrange temporary lodging near treatment center, hybrid monitoring, or inpatient initiation.
Remote monitoring false reassurance	Digital data are reviewed late or do not capture subtle neurologic change.	Use remote monitoring as an adjunct, not a substitute for symptom-triggered calls and in-person evaluation. Define who reviews alerts and response time expectations.
Incomplete handoff after clinic discharge	After-hours team lacks dose timing, baseline neurologic status, or product-specific risk window.	Document last dose, step-up number, CRS/ICANS history, baseline ICE/handwriting, caregiver contact, and preferred ED pathway at each transition.

Failure-mode analysis should be adapted locally and reviewed after every unplanned emergency department visit, admission, grade 2 or higher CRS/ICANS, or delayed escalation event.

## Supportive care for acute and ongoing toxicities

### Cytokine release syndrome and the CRS-infection diagnostic overlap

CRS is the dominant early toxicity during step-up dosing and the main reason for structured observation. In outpatient settings, the decisive event is often not severe CRS at presentation but early fever, chills, tachycardia, malaise, or dizziness reported from home. Nursing triage must therefore respond before hypotension or hypoxia develops (4–6, 16–20).

A critical limitation of outpatient models is diagnostic ambiguity. In an immunocompromised RRMM patient, fever after a step-up dose should be approached simultaneously as possible CRS and possible infection until sufficient clinical information supports de-escalation. Prematurely assigning fever to CRS can delay antibiotics and sepsis management; prematurely assigning fever to infection can delay CRS-directed treatment. The revised framework uses a dual-track fever approach rather than a single diagnostic pathway (19, 20, 23–25), This dual-track fever assessment is summarized in (**Table 4**).

**Table 4:** Dual-track fever assessment after bispecific antibody step-up dosing .

Step	Parallel CRS and infection assessment	Nursing decision point
Trigger	Temperature >=38.0 C after step-up dose, rigors with evolving symptoms, new tachycardia, dizziness, dyspnea, hypotension, hypoxia, or caregiver-reported acute change.	Treat as urgent. Instruct patient not to self-medicate before contacting team unless directed.
Immediate assessment	Vital signs, oxygen saturation, mental status, dose timing, product and step-up number, baseline risk factors, current medications, neutropenia status, infection symptoms, line status.	Determine whether patient needs immediate ED return, direct clinic evaluation, or emergency medical services.
CRS track	Grade fever, hypotension, hypoxia, and trajectory using ASTCT concepts; assess for rapid progression; prepare IV access; notify prescriber; administer supportive care and CRS-directed therapy per protocol.	Low threshold for monitored setting when fever is recurrent, patient is frail, symptoms evolve rapidly, or product-specific risk window remains active.
Infection/sepsis track	Assess neutropenia, recent infection, cough, urinary symptoms, diarrhea, skin/line findings, cultures and labs when indicated, and need for empiric antimicrobials.	Do not delay antimicrobial therapy when sepsis, neutropenic fever, or unstable infection is possible.
Disposition decision	Outpatient observation, extended clinic monitoring, admission, ICU transfer, or emergency services based on physiologic status, distance from care, caregiver reliability, and product-specific risk.	If uncertainty persists, choose the safer disposition. Outpatient monitoring should not continue when communication or transport is unreliable.

This table adapts ASTCT CRS concepts and MM BsAb toxicity guidance for outpatient triage. It is a practice framework and not a validated diagnostic algorithm.

Initial nursing actions include immediate vital signs, oxygen saturation, symptom review, focused neurologic check, confirmation of dose timing, assessment of neutropenia and infection risk, review of central lines and localizing symptoms, and rapid notification of the prescribing team. Cultures, imaging, empiric antimicrobials, intravenous fluids, tocilizumab, corticosteroids, and admission are determined by institutional protocols and physician/advanced-practice assessment. Biomarkers such as C-reactive protein, ferritin, interleukin-6, or procalcitonin may provide context but should not be used in isolation to distinguish CRS from infection.

### ICANS and subtle neurotoxicity

ICANS is less frequent than CRS with currently approved MM BsAbs, but outpatient recognition is challenging because early manifestations may be subtle and may be noticed first by caregivers. Nurses should compare each assessment with the patient’s documented baseline rather than relying only on obvious confusion (7–10, 19, 20).

Symptoms requiring urgent reassessment include word-finding difficulty, dysgraphia, impaired attention, slowed responses, new somnolence, tremor, gait instability, behavioral change, headache with cognitive change, aphasia, focal weakness, seizure, or caregiver concern that the patient is not acting normally. Baseline handwriting, orientation, attention testing, and an ICE or locally standardized neurologic assessment improve detection. Fall precautions, aspiration precautions, withholding subsequent doses, and urgent escalation are appropriate when new neurologic symptoms emerge. Because isolated ICANS may not respond to tocilizumab, nursing teams must be familiar with corticosteroid-based and neurologic evaluation pathways (19, 20, 23).

### Infection risk, hypogammaglobulinemia, and prolonged immune dysfunction

Infection prevention is not limited to the step-up window. BsAb therapy is delivered to patients with RRMM who often have cumulative immune injury from prior therapy, marrow compromise, prior anti-CD38 exposure, prior BCMA-directed therapy or CAR T-cell therapy, repeated corticosteroids, lymphopenia, neutropenia, and impaired humoral immunity. BsAbs may deepen or prolong this immune dysfunction through plasma-cell depletion, hypogammaglobulinemia, impaired vaccine responses, and altered T-cell function (24, 26–31).

The revised framework treats infection monitoring as a longitudinal nursing responsibility. Baseline review should include recent infections, antimicrobial exposure, antiviral and Pneumocystis jirovecii pneumonia prophylaxis plans where indicated, immunoglobulin levels, vaccination status, neutrophil and lymphocyte trends, and institutional criteria for IVIG. During therapy, nurses should monitor for recurrent sinopulmonary infections, opportunistic infections, persistent viral reactivation, infection-related treatment delays, need for IVIG, and adherence to prophylactic regimens. Fever during step-up dosing requires dual-track evaluation, but infection vulnerability persists after CRS risk declines (24, 26–31).

A forward-looking outpatient program should also anticipate emerging complications. As patients remain on BsAbs for prolonged periods or receive BsAbs after prior T-cell-directed therapy, centers should track late infection patterns, cumulative hypogammaglobulinemia, antimicrobial resistance, impact of dose-spacing strategies, vaccine responsiveness, and patient adherence to prevention behaviors. These outcomes should be incorporated into program evaluation rather than handled as isolated adverse events.

### Cytopenias and hematologic supportive care

Baseline marrow compromise is common in heavily pretreated RRMM, and BsAbs may exacerbate neutropenia, anemia, and thrombocytopenia. Nurses should review complete blood count trends before each dose, assess for bleeding, symptomatic anemia, fever, and functional decline, and coordinate transfusion, growth factor support, antimicrobial evaluation, and treatment delays according to institutional protocols (4–10, 16–18, 24, 26, 27).

Cytopenia management also intersects with outpatient feasibility. Severe neutropenia increases the danger of misclassifying fever as CRS alone; thrombocytopenia and anemia may reduce physiologic reserve during hypotension or infection; and transfusion dependence may increase visit complexity. Therefore, cytopenias should be incorporated into pre-dose readiness and not treated only as laboratory abnormalities.

### Agent-specific toxicities: nursing implications for talquetamab

GPRC5D-directed therapy creates a distinctive toxicity profile that can affect nutrition, function, and quality of life beyond the acute CRS window. Nurses should monitor dysgeusia, ageusia, xerostomia, dysphagia, oral discomfort, weight loss, xerosis, desquamation, rash, nail fragility, fissuring, pain, and limitations in activities of daily living (6, 9, 18, 32).

Management should be anticipatory. Practical strategies include baseline weight and nutrition assessment, early dietitian referral, high-calorie or texture-modified diet plans, oral care routines, saliva substitutes, avoidance of irritating foods, dermatologic evaluation for persistent rash or skin breakdown, nail protection, foot and hand care, and close tracking of weight and intake. These measures are not cosmetic adjuncts; they can determine treatment continuity when taste alteration, oral symptoms, or skin/nail toxicity threaten adherence and functional status (9, 18, 32).

## Patient and caregiver education

Education should begin before the first step-up dose and continue across the initiation period. The content must be specific enough to support action under stress. Patients and caregivers should understand why step-up dosing is used, what fever threshold matters, why fever must be reported rather than self-treated, which neurologic and respiratory symptoms require urgent contact, whom to contact after hours, and how transportation will occur if escalation is required (7–10, 14, 33–35).

Teach-back is essential. Rather than asking whether the patient understands, nurses should ask the patient and caregiver to demonstrate thermometer technique, state the fever threshold, name at least three red-flag symptoms, identify the 24-hour contact number, explain when to call emergency services, and describe how they will reach the treatment center at night. Educational materials should be written in plain language and translated when needed. Cultural factors should also be addressed: in some settings, patients may delay reporting because they do not want to trouble the team. Nurses should explicitly frame early reporting as a required safety behavior rather than an overreaction (33–35). The essential discharge teaching bundle is summarized in **Box 1**.

Box 1 **Essential discharge teaching bundle for step-up dosing**

•Monitoring: verify thermometer technique; define frequency and duration of temperature and symptom checks; provide a symptom diary or remote reporting tool when used.

•Red flags: fever >=38.0 C, rigors, dizziness, shortness of breath, oxygen desaturation, confusion, speech change, word-finding difficulty, tremor, unusual sleepiness, gait change, inability to maintain intake, or caregiver concern.

•Action plan: provide one 24/7 contact pathway; specify when to call the oncology team, return to the treating center, proceed to the emergency department, or activate emergency medical services.

•Medication cautions: instruct patients not to self-manage suspected post-dose fever with antipyretics unless the oncology team directs them to do so.

•Caregiver confirmation: document that the caregiver can provide continuous observation during the risk window, perform teach-back, and mobilize transport.

•Emergency communication: provide a wallet card or letter naming the BsAb, most recent dose date/time, CRS/ICANS risk, treating center contact, and recommended ED escalation pathway.

## Multidisciplinary coordination and regional implementation

Safe outpatient step-up dosing requires coordination among nurses, hematologists, advanced practice providers, pharmacists, infusion services, after-hours triage teams, emergency departments, inpatient units, intensive-care teams, infectious disease specialists, neurologists, dermatologists, dietitians, social workers, and caregivers. Nurses frequently function as the operational hub by verifying readiness, identifying risk changes before dosing, monitoring early symptoms, reinforcing education, and ensuring that handoffs contain the information needed for rapid escalation (11, 14, 20, 22).

The revised manuscript emphasizes that outpatient step-up dosing is not a portable intervention. A pathway that is safe in a high-volume tertiary myeloma program may be unsafe in a center without immediate hematology backup or an emergency department familiar with immune-effector toxicity. Institutions should explicitly choose one of several resource-stratified models: full inpatient initiation, hybrid initiation with selected outpatient components, near-center ambulatory initiation with temporary lodging, or highly selected outpatient initiation with robust after-hours pathways. These resource-stratified implementation models are summarized in (**Table 5**).

**Table 5:** Resource-stratified implementation models for BsAb step-up dosing .

Model	Appropriate context	Advantages	Limitations
Full inpatient initiation	Limited after-hours infrastructure; remote geography; high patient risk; no reliable caregiver; unfamiliar ED; label or local policy requiring hospitalization.	Highest direct monitoring, rapid therapy access, easier neurologic comparison.	Higher bed use, patient burden, cost, and potential delays if beds unavailable.
Hybrid initiation	Patient or product risk is intermediate; center can provide clinic dosing but uses planned overnight or short-stay monitoring after selected doses.	Balances bed use and safety; adaptable to product-specific windows.	Requires precise scheduling, handoff, and criteria for which doses require admission.
Near-center outpatient initiation	Patient is stable but lives far away; temporary lodging or observation unit is available near the treatment center.	Reduces inpatient days while preserving rapid access.	Cost and caregiver burden may be substantial; lodging is not a substitute for clinical monitoring.
Fully outpatient initiation	Low-risk patient; reliable caregiver; short travel time; 24/7 trained triage; ED protocol; rapid access to tocilizumab, corticosteroids, admission, and ICU.	Potentially reduces hospitalization and patient disruption.	Narrow margin for error; unsuitable if communication, transport, or rescue capacity is uncertain.

Local policies and product labels may require hospitalization regardless of institutional preference. The safest model is the one aligned with patient biology, product risk, caregiver reliability, and rescue capacity.

## Asia-Pacific considerations

The Asia-Pacific region is heterogeneous with respect to tertiary cancer-center density, geography, health financing, emergency access, language diversity, and caregiver structures. Published regional experience specific to BsAb implementation remains limited, although emerging Japanese and Chinese talquetamab data suggest safety patterns broadly consistent with global experience (36–38).

Regional implementation should therefore move beyond acknowledging heterogeneity and explicitly match care models to infrastructure. Metropolitan tertiary centers with experienced hematology teams, infusion capacity, remote triage, and rapid inpatient transfer may support selected outpatient or hybrid models. Rural or resource-limited settings may require inpatient initiation, referral to specialized centers for step-up dosing, or temporary accommodation near the treating hospital. Language-concordant education, culturally responsive communication, and family-capacity assessment are particularly important. In settings where patients hesitate to report symptoms because they fear burdening clinicians, nurses should repeatedly state that early reporting of fever or neurologic change is a mandatory component of safe therapy (14, 15, 34–36, 39).

## Continuous evaluation, digital health, and nursing-sensitive outcomes

Outpatient BsAb programs should be learning systems. The original manuscript listed nursing-sensitive outcomes; the revised framework embeds them in a continuous quality-improvement process. Centers implementing outpatient or hybrid step-up dosing should define a minimum dataset before launch, review outcomes after each toxicity event, and update eligibility criteria, discharge instructions, observation windows, and emergency pathways through iterative cycles (40, 41).

Digital symptom reporting, wearable temperature or oxygen monitoring, automated reminders, and telehealth follow-up may improve oversight but should be evaluated carefully. Technology can reduce information gaps only if alerts are reviewed in real time, escalation responsibilities are explicit, and patients can still access urgent in-person care. Programs should avoid using remote monitoring to justify outpatient initiation when transport, caregiver reliability, or emergency access is inadequate.

The minimum dataset should include both safety outcomes and implementation outcomes. Safety outcomes include CRS and ICANS grade, infection, antimicrobial use, IVIG, cytopenia support, ED visits, admission, ICU transfer, and treatment interruption. Implementation outcomes include eligibility decision fidelity, teach-back completion, caregiver confirmation, follow-up call completion, time from symptom onset to patient call, time from call to clinician response, time to in-person assessment, handoff completeness, remote-monitoring adherence, patient confidence, caregiver burden, and equity indicators such as travel distance, language needs, and temporary lodging access. A proposed minimum dataset for continuous evaluation is provided in **Box 2**.

Box 2 **Minimum dataset for continuous evaluation of outpatient or hybrid BsAb step-up programs**

•Eligibility and readiness: documented multidisciplinary assessment, risk-domain review, baseline neurologic assessment, infection review, immune status, caregiver confirmation, and transport plan.

•Monitoring fidelity: premedication completion, observation duration, vital-sign schedule, follow-up calls, remote-monitoring alerts, written discharge education, and wallet-card provision.

•Escalation timeliness: time from symptom onset to patient/caregiver call, call to clinician response, triage decision to evaluation, and evaluation to CRS- or infection-directed therapy.

•Acute safety: grade 2 or higher CRS, any ICANS, recurrent fever, hypoxia, hypotension, ED visit, unplanned admission, ICU transfer, and mortality during the step-up window.

•Longitudinal supportive care: serious or recurrent infections, neutropenia, transfusion, growth factor use, IVIG, antimicrobial prophylaxis adherence, talquetamab-related weight loss, dietitian/dermatology referral, and treatment interruption.

•Patient and caregiver experience: teach-back success, confidence in escalation instructions, caregiver burden, missed monitoring steps, language needs, travel burden, temporary lodging use, and financial toxicity.

•Learning process: case review after each failure event, pathway revision log, staff education updates, and periodic reporting to the multidisciplinary myeloma program.

## Controversies, evidence gaps, and future directions

The evidence base for outpatient BsAb step-up dosing remains incomplete. Current practice is still shaped primarily by product labels, single-arm safety reports, expert consensus, infection recommendations, and limited institutional implementation reports rather than prospective comparative trials. The absence of validated outpatient eligibility criteria is a major gap (7–11, 14, 15, 19, 20).

Several uncertainties require prospective study. First, the field needs product-specific and resource-stratified evidence rather than pooled outpatient claims across different BsAbs. Second, patient-selection models should incorporate measurable disease biology, immune reserve, frailty, and social determinants of safe care. Third, the CRS-infection overlap requires pragmatic algorithms that minimize both delayed antibiotics and delayed CRS-directed therapy. Fourth, remote monitoring should be tested against clinically meaningful outcomes such as time to escalation, unplanned admission, safety, patient burden, and equity. Fifth, the impact of prolonged BsAb exposure on immune architecture, infection risk, vaccination response, and T-cell exhaustion requires longer follow-up.

Future nursing research should evaluate whether standardized teach-back, caregiver certification, wallet cards, after-hours scripts, ED handoff templates, and prospective quality datasets reduce failure events. Importantly, outpatient initiation should not be judged successful only by reduced hospital days. It should be judged by safe toxicity recognition, timely intervention, preserved treatment continuity, patient and caregiver confidence, and equitable access across geography and health systems.

## Discussion

Outpatient step-up dosing of BsAbs sits at the intersection of therapeutic innovation, immune-effector toxicity, nursing surveillance, and health-system capacity. The revised manuscript reframes this practice as a biologically informed and failure-aware care model. The main question is not whether selected patients can receive step-up doses outside the hospital, but how institutions can identify the subset for whom the combined patient-caregiver-system unit can detect and reverse toxicity in time.

Several conclusions follow. Product-specific monitoring is essential because BsAbs differ in timing and pattern of toxicity. Patient selection must integrate disease biology and immune reserve rather than relying solely on logistics. Fever must be evaluated through a dual CRS-infection lens because diagnostic certainty is often unavailable at first contact. ICANS surveillance must focus on subtle deviation from baseline. Infection prevention must extend beyond early dosing and account for cumulative immune dysfunction. Finally, outpatient programs must be continuously evaluated using nursing-sensitive and implementation outcomes rather than static checklists.

This review has limitations. It is a structured narrative synthesis and not a systematic review or meta-analysis. The framework is based on prescribing information, published safety data, consensus guidance, and implementation reports, and the proposed selection domains and monitoring pathways require prospective validation. Regulatory requirements vary by jurisdiction and may change over time. Moreover, evidence from high-volume centers may not generalize to community, rural, or resource-limited settings.

Despite these limitations, a more critical and biologically integrated framework is necessary as BsAbs move into broader practice. Outpatient step-up dosing should not be used as a default marker of modernization or efficiency. It should be reserved for circumstances in which product kinetics, patient biology, caregiver performance, and institutional rescue capacity are aligned. When these conditions are not met, inpatient or hybrid initiation remains the safer and more scientifically defensible model.

## Data Availability

No new datasets were generated or analyzed in this study.
